# CAR Modulates E-Cadherin Dynamics in the Presence of Adenovirus Type 5

**DOI:** 10.1371/journal.pone.0023056

**Published:** 2011-08-05

**Authors:** Fawziyah Hussain, Penny E. Morton, Marjolein Snippe, Janis Sullivan, Charlotte Farmer, Marisa L. Martin-Fernandez, Maddy Parsons, George Santis

**Affiliations:** 1 Division of Asthma, Allergy and Lung Biology, King's College London, London, United Kingdom; 2 Science and Technology Facilities Council, Rutherford Appleton Laboratory, Didcot, Oxford, United Kingdom; 3 Randall Division of Cell and Molecular Biophysics, King's College London, London, United Kingdom; French National Centre for Scientific Research, France

## Abstract

Adenovirus (Ad) serotype 5 (Ad5) fiber competitively binds to the coxsackievirus and Ad receptor (CAR) to attach Ad5 to target cells and also disrupts cell junctions and facilitates virus escape at a late stage in Ad5 infection. Here we demonstrate that paracellular permeability in MCF7 and CAR overexpressing MCF7 (FLCARMCF7) cells is increased within minutes following the addition of Ad5 to cells. This is brought about, at least in part, by altering the molecular dynamics of E-cadherin, a key component of the cell-cell adhesion complex. We also demonstrate that the increase in E-cadherin mobility is constitutively altered by the presence of CAR at FLCARMCF7 cell junctions. As increased paracellular permeability was observed early after the addition of Ad5 to cells, we postulate that this may represent a mechanism by which Ad5 could disrupt cell junctions to facilitate further access to its cell receptors.

## Introduction

Most Adenovirus (Ad) species attach to host cells through the interaction of the fiber protein with the Coxsackievirus and Adenovirus (Ad) receptor (CAR) [1], [2], [3], [4]. CAR is a transmembrane protein that is predominantly localised to the baso-lateral surface of polarised epithelium and at tight junctions where it binds to multiple proteins including zonula occludens (ZO-1), β-catenin [5], [6], [7], [8], actin [9] and tubulin [10]. CAR is one of a number of immunoglobulin-like molecules at cell junctions. These include junctional adhesion molecules (JAM)-A, B & C [11], [12], [13], [14]; endothelial cell-selective adhesion molecule (ESAM) [15]; and JAM4 [16]. As a component of cell junctions, CAR may mediate cell adhesion by virtue of homophilic interaction between CAR molecules on adjacent cells. Interestingly, Ad serotype 5 (Ad5) fiber protein binds to CAR with higher affinity than CAR does for itself [17] suggesting that Ad5 may weaken cell junctions by disrupting CAR interaction between adjacent cells. Ad5 exploits its ability to competitively bind to CAR to disrupt junctions to escape infected cells and further propagate infection [18]. This is brought about by excess fiber, produced at the late stage of Ad5 infectious cycle. This disruption in cell junctions was shown to coincide with re-localisation of -catenin, which in fact co-immunoprecipitates with CAR not only in A549 [18] but also Sertoli cells [19]. This led to the suggestion that Ad5 disruption of cell junctions at the late stages of its infectious cycle is independent of E-cadherin. E-cadherin is however a key component of the cell adhesion complex [20], [21] where it promotes cell-cell contact through homophilic binding to E-cadherin molecules on adjacent cell junctions supported by actin filaments through β- and α-catenins [22], [23], [24], [25]. It is generally accepted that E-cadherin is highly mobile at immature and developing cell contacts, but in mature junctions, the mobile pool of E-cadherins is in the minority [22], [23]. For example, in new areas of cell contact, E-cadherin pool is mainly composed of a highly mobile fraction (90%). Once E-cadherin clusters are formed and E-cadherin begins to interact with the cytoskeleton, a much smaller fraction (50%) remains mobile. At mature junctions, the mobile fraction is even smaller (<10%) [22], [23]. The mobile E-cadherin population is monomeric; it diffuses on the membrane, exchanges with the stable E-cadherin population at contact sites and is not involved in cell adhesion [26]. Regulation of E-cadherin levels in developing and mature junctions is a dynamic process that is not fully understood. Recent evidence suggests that recycling of E-cadherin at cell junctions involves exchange between membrane and intracellular pools of E-cadherin, a process linked to endocytosis [27].

As Ad5 binds to CAR with similar affinity as does soluble Ad5 fiber [4], we hypothesised that intact Ad5 virion may disrupt cell junctions and alter paracellular permeability at an early stage in its infectious cycle, not just at late stages, as it was previously shown. We addressed this question in MCF7 cells that are a human breast tumour-derived cell line and had been extensively used to investigate the regulation of cell junctions and more specifically E-cadherin dynamics during junction formation and disruption [27], . Moreover, we have already shown that MCF7 cells have low endogenous CAR levels, express αvβ3 integrin that mediates Ad5 internalisation and are infectable by Ad5 [31]. We have also shown that CAR-RFP when expressed on the cell surface of FLCARMCF7 cells localises at cell junctions and efficiently facilitates Ad5 infection [31]. In the same study, we also showed no difference in proliferation rate between MCF7 and FLCARMCF7 cells and went on to demonstrate that signalling downstream of CAR can have effects on integrins and CAR itself to promote Ad5eGFP binding [31].

Our experiments demonstrate that high viral loads of Ad5GFP (Ad5eGFP is an E1–E3-deleted Ad5 that expresses green fluorescent protein (GFP) as a transgene under the control of the cytomegalovirus promoter) increased paracellular permeability in MCF7 and CAR over-expressing MCF7 cell junctions. We also demonstrate enhanced E-cadherin molecular dynamics in the presence of Ad5GFP, a process that appeared to be modulated by CAR.

## Results

### High CAR expression leads to reduced E-cadherin at cell-cell junction

**Figure 1:**
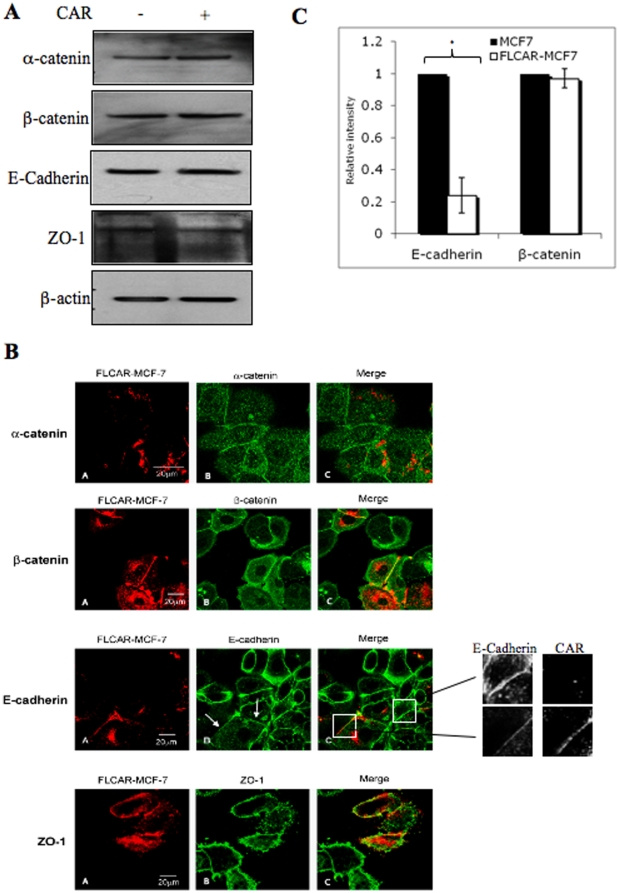
CAR reduces E-cadherin expression at CAR-expressing MCF7 cell junctions. (**A**) MCF7 parental (−) or FLCARMCF7 (+) cell lysates subjected to western blot analysis for total α-catenin, β-catenin, E-cadherin and ZO-1 protein levels. Actin serves as a loading control. There was no difference in the level of expression of these proteins in the presence/absence of CAR. (**B**) Confocal images of MCF7 and FLCARMCF7 (red channel) cells immunostained for α-catenin, β-catenin, E-cadherin and ZO-1 antibodies labelled with Oregon green. The levels and localisation of α-catenin, β-catenin and ZO1 appeared the same in MCF7 and FLCARMCF7 cells but E-cadherin expression was reduced in CAR expressing junctions (denoted by white arrows and highlighted in the inset). These are representative images from at least 3 experiments, with reduced E-cadherin being evident in more than 80% of CAR expressing junctions. (C) Bar charts are quantitations of E-cadherin or β-catenin intensity at junctions between FLCARRFP positive cells (FLCARMCF7), or those without CAR (MCF7) and calibrated on a per pixel basis to correct for any differences in junction size/area. MCF7 junction intensity values were normalised to 1 and all values for FLCARMCF7 junctions represented as a relative value to this. Values were pooled from multiple cells and images (n = >25 junctions per condition) over three independent experiments and represented as relative mean intensity * P<0.05. Significance was determined by a two-way anova.

Junctional membrane proteins and E-cadherin in particular play a key role in cell-cell junction formation and stability. As CAR is a transmembrane cell-cell junction, protein we assessed the effect of its over expression on other junctional membrane proteins. We therefore compared endogenous levels of E-cadherin, β-catenin, α-catenin and ZO-1 between MCF7 cells and MCF7 cells stably expressing functional full-length human CAR C-terminally tagged with monomeric red fluorescent protein (FLCARRFP; FLCARMCF7 cells) [31] to determine if endogenous levels of these proteins were affected by the over-expressed CAR. We had previously reported that FLCARRFP is efficiently recruited to junctions in MCF7 cells [31] (Supplementary Figure S1). Figure 1 shows that expression levels of β-catenin, α-catenin and ZO-1 were equal between MCF7 and FLCARMCF7 cells (Figure 1A). To then assess if the localisation of these proteins was altered by the over-expression of CAR, confocal images were also taken of a 1∶1 mixed population of MCF7 and FLCARMCF7 cells. Images in Figure 1B demonstrate that α-catenin, β-catenin and ZO-1 localise similarly to FLCARMCF7 and MCF7 cell junctions. In contrast, the level of E-cadherin present at CAR positive FLCARMCF7 cell junctions appeared to be consistently lower compared to MCF7 cell junctions (Figure 1B). To evaluate this further, intensity line scans were taken across individual junctions highlighted in Figure 1B and the relative levels of CAR and E-cadherin assessed using ImageJ and intensity profiles plotted over distance. Intensity levels of E-cadherin at junctions between multiple FLCARRFP positive cells, or those without FLCARRFP was then quantified and shown to be significantly greater in MCF7 compared to FLCARMCF7 cell junctions (Figure 1C). This data indicates that E-cadherin localisation to cell junctions is reduced in the presence of overexpressed CAR.

**Figure 2:**
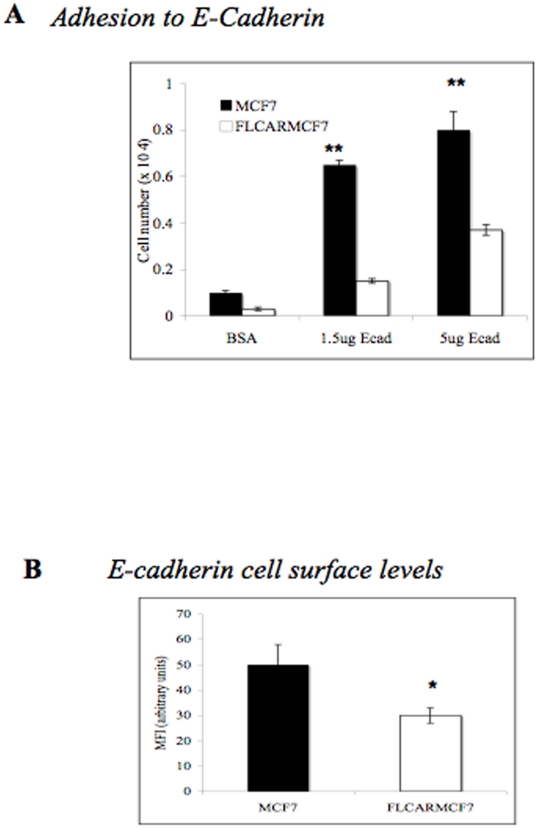
CAR overexpression reduces E-cadherin cell adhesion and E-cadherin cell surface levels. (**A**) E-cadherin extracellular domain was immobilised onto 24-well plates at 1.5 µg/ml or 5 µg/ml. BSA was used as a control. MCF7 (black bars) or FLCARMCF7 cells (white bars) were seeded at 5×10^4^/well, left for 2 hours, and adherent cells counted. Error bars depict SEM (n = 4). ** p<0.01. (**B**) FACS analysis of E-cadherin cell-surface expression on MCF7 (black bar) and FLCARMCF7 cells (white bar). MFI per cell pooled from 4 independent experiments is shown +/−SEM. * P<0.05. Significance was determined by a two-way anova.

In order to obtain independent confirmation that CAR over expression was associated with reduced E-cadherin in FLCARMCF7 cells, a cell-binding assay was then conducted to assess the level of binding of FLCARMCF7 cells to immobilised purified extracellular domain of E-cadherin. Figure 2A shows significantly higher binding of MCF7 cells to the E-cadherin extracellular domain compared to FLCARMCF7 cells. To further confirm these findings, we used FACS to analyse cell surface levels of E-cadherin in both cell lines. Figure 2B demonstrates a significant reduction in cell surface levels of E-cadherin in FLCARMCF7 compared to parental cells. Taken together, data based on microscopy experiments, cell binding assays and FACS analysis, suggest that high levels of CAR can alter E-cadherin cell surface levels on FLCARMCF7 cells and at FLACRMCF7 cell-cell junctions.

### Paracellular permeability is increased in the presence of Ad5

**Figure 3:**
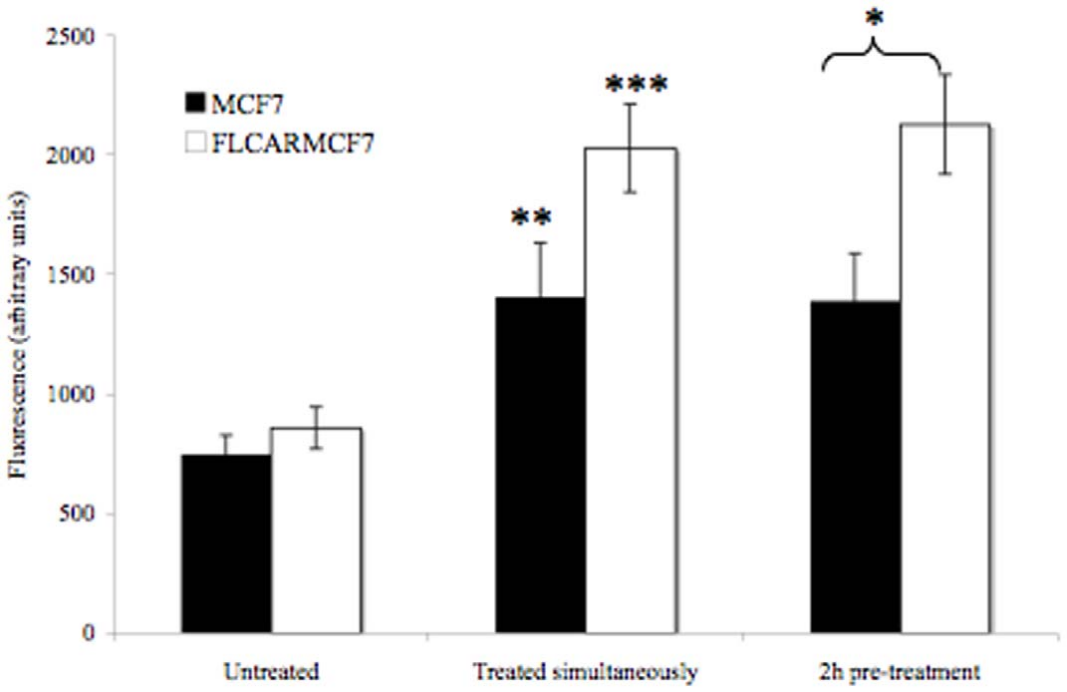
Ad5eGFP increases paracellular permeability. Paracellular permeability was assessed across MCF7 and FLCARMCF7 either basally and in response to Ad5eGFP (9000 VP/cell) added either simultaneously or 2 hours prior to incubation with FITC-dextran (1 mg/ml). No difference in basal permeability was seen between MCF7 and FLCARMCF7 cells. Incubation with Ad5eGFP led to increased permeability in MCF7 and FLCARMCF7 cells and this increase was more pronounced in FLCARMCF7 cells. *** = p<0.001, ** = p<0.01, * = p<0.05.

Since CAR expression reduced E-cadherin cell surface levels and also E-cadherin levels at FLCARMCF7 cell junctions, we asked whether this had an effect on paracellular permeability. We found no difference in basal permeability between MCF7 and FLCARMCF7 cells (Figure 3). This suggests that the reduced E-cadherin level at FLCARMCF7 cell junctions had no functional consequence on the integrity of cell-cell contacts. One possible explanation for this observations is that CAR-CAR interactions contributes to the maintenance of cell-cell junctions in FLCARMCF7 cells and thus compensates for the reduced E-cadherin levels in these cells. We also found that the addition of Ad5eGFP increased paracellular permeability in MCF7 and FLCARMCF7 cells (Figure 3) and that this increase was more pronounced in FLCARMCF7 cells than the parental cells. As Ad5eGFP is replication deficient, disruption of cell junctions is likely to be a function of the intact Ad5eGFP virion and not a consequence of excess soluble fiber produced after virus replication, at a late stage in the virus life cycle.

### E-cadherin de-stabilises at cell-cell junctions in the presence of Ad5eGFP

**Figure 4:**
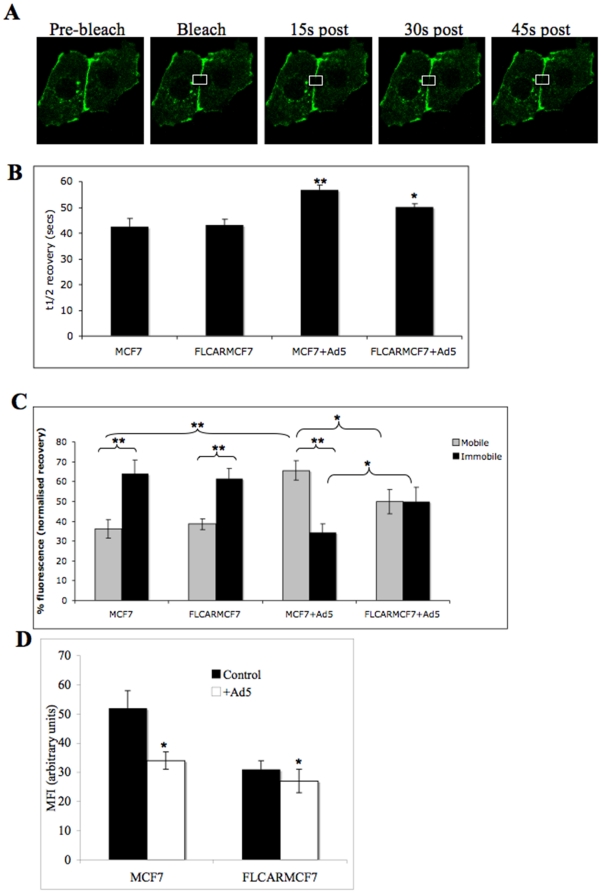
E-cadherin is more dynamic within junctions in the presence of Ad5eGFP. (**A**) Example images from a FRAP experiment on MCF7 cells expressing E-cadherin-GFP. MCF7 were transiently transfected with E-cadherin-GFP for 24 hours before being plated on glass bottomed imaging chambers. Images were captured, analysed and exported using NIS Elements AR software. An image was taken immediately after the bleach and one 5 seconds after that, and then images were taken every 15 seconds for 5 minutes. Images shown for pre-bleach, bleach (bleached region shown in white box) and the recovery of E-cadherin-GFP monitored at 15 s, 30 s and 45 s post-bleach. (**B**) The half-life (τ1/2) of E-cadherin recovery after photobleaching MCF7 and FLCARMCF7 cells in the absence or presence of Ad5eGFP (MCF7+Ad5 and FLCARMCF7+Ad5 respectively). E-cadherin recovery was defined from corrected intensity data fitted to a single exponential equation and calculated using the equation *t*_1/2_ = ln 0.5/−τ. Data was pooled from >12 cells per condition over 4 independent experiments +/− SEM. Significance was determined by two-way anova. ** = p<0.001 compared to MCF7 cells no Ad5eGFP; * = p<0.05 compared to FLCARMCF7 no Ad5eGFP; * = p<0.05 compared to FLCARMCF7 with Ad5eGFP. (**C**) Mobile and immobile E-cadherin fractions in MCF7 and FLCARMCF7 cells in the absence or presence of Ad5eGFP (MCF7+Ad5 and FLCARMACF7+Ad5 respectively). Mobile and immobile fractions were defined as the percentage recovery at plateau (mobile) and remaining non-recovered fraction at this time (immobile fraction). Significance was determined by two-way anova. * * = P<0.001 * = P<0.05. (**D**) FACS analysis of cell surface E-cadherin levels on MCF7 and FLCARMCF7 cells without (black bars) or with Ad5eGFP (9000 VP/cell; white bars). MFI is shown pooled from 3 independent experiments +−/SEM. Significance was determined by a two-way anova. * = p<0.05 compared to MCF7 no Ad5eGFP levels.

We then analysed the effect of Ad5eGFP on E-cadherin mobility by determining the GFP tagged E-cadherin (E-cadherin-GFP) fluorescence recovery after photobleaching (FRAP) MCF7 and FLCARMCF7 cell junctions. Increased ability of E-cadherin to move at junctions would be reflected by an increase in FRAP [22]. MCF7 and FLCARMCF7 cells were transiently transfected with E-cadherin-GFP and the rate of recovery of this protein was examined every 15 seconds post bleach for 5 minutes (Figure 4A). Under steady state conditions, the half-life (τ1/2) of recovery, which measures the rate of E-cadherin recovery (Figure 4B), and the mobile and immobile E-cadherin fractions, which measures the amount of fluorescent E-cadherin present in the bleach region at the end of the experiment (Figure 4C), were similar in MCF7 and FLCARMCF7 cells. The immobile E-cadherin fraction was in the majority in both cell types (Figure 4C). E-cadherin maximal percentage recovery was essentially the same as the percentage recovery of the E-cadherin mobile fraction (Figure 4C). In MCF7 cells the presence of Ad5eGFP increased the half-life of E-cadherin recovery indicating that Ad5eGFP leads to a reduction in the speed of recruitment of E-cadherin molecules to MCF7 cell junctions. Ad5eGFP also increased the proportion of E-cadherin in the mobile fraction, indicating an increase in the mobility of E-cadherin. This demonstrates that Ad5eGFP leads to both increased translocation of E-cadherin away from, and decreased rate of E-cadherin recruitment to MCF7 cell junctions. This could result in a net reduction in E-cadherin at MCF7 cell junctions. In support of this, FACS analysis showed that Ad5eGFP significantly reduced E-cadherin cell surface levels in MCF7 cells (Figure 4D). The presence of FLCARRFP in FLCARMCF7 cell junctions did not alter the E-cadherin mobility in the absence of Ad5eGFP. However, the addition of Ad5eGFP to FLCARMCF7 cells led to more immobile E-cadherin being maintained at FLCARRFP containing cell junctions compared to MCF7 cell junctions (Figure 4C). This suggests that the Ad5eGFP-driven increase in E-cadherin mobility is reduced in the presence of overexpressed CAR. Interestingly, in FLCARMCF7 cell junctions we observed slower rates of E-cadherin recovery in the presence of Ad5eGFP, although this rate of recovery was faster in these compared to MCF7 cells (Figure 4C). This would indicate that in cell junctions where CAR is overexpressed, the addition of Ad5eGFP results in faster turnover of E-cadherin to these junctions, where it is more likely that it will be retained as immobile fraction. This is further supported by data demonstrating that Ad5eGFP did not induce changes in surface levels of E-cadherin in FLCARMCF7 cells (Figure 4D). Taken together these data suggest that Ad5eGFP reduced E-cadherin localisation to cell junctions by modulating the dynamics of E-cadherin recruitment to junctions, and that overexpression of CAR disrupts this.

We next applied similar approaches to determine whether Ad5eGFP altered CAR levels and localisation. FACS and imaging data demonstrated no difference in CAR levels at FLCARMCF7 cell junctions in the presence of Ad5eGFP (performed in separate experiment; data not shown). Similarly, FRAP analysis to define the molecular dynamics of CAR at FLCARMCF7 cell junctions demonstrated no significant change in the rate of CAR recovery in the presence or absence of Ad5eGFP (data not shown). This would suggest that Ad5eGFP has little or no influence in the localisation and mobility of CAR at FLCARMCF7 cell junctions.

### E-cadherin/β-catenin complex is unaffected by over-expression of CAR or Ad5eGFP in MCF7 cells

**Figure 5:**
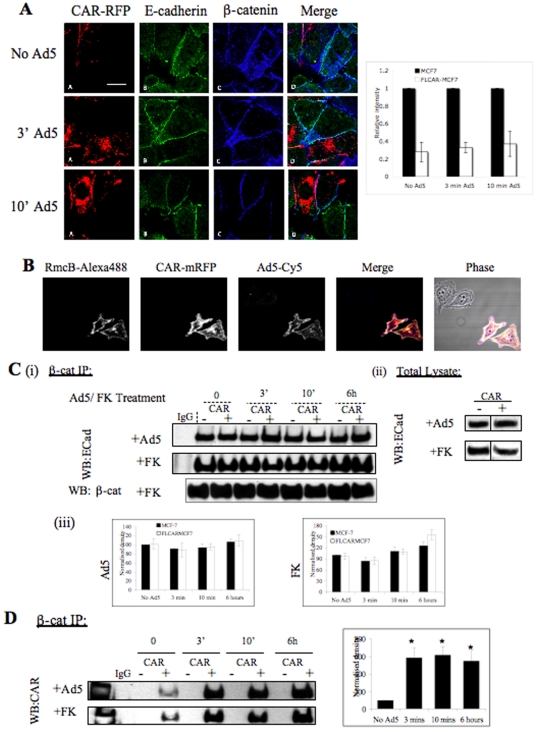
Ad5eGFP modulates CAR association with β-catenin. (A) (i) A mixed population of MCF7 and FLCARMCF7 cells were plated onto vitronectin-coated coverslips and incubated with Ad5eGFP at 9000 VP/cell for 3 minutes and 10 minutes before fixation. Cells were then immunostained for the .expression and localisation of E-cadherin (AB green) and β-catenin (AC; blue). Merged images are also shown (AD). Scale bars are 20 µm. Representative images are shown from 3 independent experiments with similar results. (ii) Bar charts are quantitations of E-cadherin intensity at junctions between FLCARRFP positive cells (FLCARMCF7), or those without CAR (MCF7) and calibrated on a per pixel basis to correct for any differences in junction size/area. MCF7 junction intensity values were normalised to 1 and all values for FLCARMCF7 junctions represented as a relative value to this. Values were pooled from multiple cells and images (n = >25 junctions per condition) over three independent experiments and represented as relative mean intensity. p>0.05 for E-cadherin in FLCARMCF7 *vs* MCF7 at all time points. (B) Fluorescence images demonstrating Ad5eGFP attachment to MCF7 and FLCARMCF7 cells. A mixed population of MCF7 and FLCARMCF7 cells were seeded onto vitronectin-coated coverslips. Cells were incubated with Ad5eGFP-Cy5 (9000 VP/cell) for 3 minutes at 37°C before fixation with 4% PFA and imaged by confocal microscopy. First two panels from left show FLCARMCF7 cells fixed and stained for CAR using RmcB (CAR-specific antibody) [1] followed by an Alexa fluor 488-conjugated secondary antibody (first panel) and RFP. Third panel shows Ad5eGFP-Cy5 attachment to cells and fourth panel from left shows merged image indicating FLCARMCF7 cells to which Ad5eGFP–Cy5 was bound. Ad5eGFP-Cy5 faint binding to MCF7 cells is also shown in the third and fourth panels. MCF7 cells in these panels are identified by lack of CAR or RFP expression. (C) (i) MCF7 and FLCARMCF7 cells were incubated with Ad5eGFP (+Ad5) at 9000 VP/cell or FK (+FK) at 160 µg/ml for 3 min, 10 min or 6 hours. Cells were then lysed and immuno-precipitated with β-catenin or control IgG followed by probing for E-cadherin. Blots were re-probed with β-catenin to control for loading and β-catenin re-probe of FK experiment is shown as an example. (ii) Whole cell lysates used for the β-catenin IP, run on a separate gel and shown for presentation. (iii) Bar charts showing mean densitometry quantification +/− SEM from four independent experiments showing relative levels of the E-cadherin/β-catenin complex for each Ad5 and FK treatments. (D) Blots as in experiments outlined in (B) re-probed for the presence of CAR. Bar chart is mean densitometry quantification +/− SEM from four independent experiments showing relative levels of CAR within the complex for Ad5 treatment. Significance was determined by a one-way anova. * = P<0.05.

Maintenance of E-cadherin at cell junctions is partly controlled by interaction of its cytoplasmic domain with β-catenin [24], [25]. Therefore we investigated whether the localisation of the E-cadherin/β-catenin complex was affected by the over-expression of CAR or addition of Ad5eGFP. First, a mixed population of MCF7 and FLCARMCF7 cells were incubated with Ad5eGFP for 3 and 10 minutes at 37°C. These time points coincide with Ad5 cell attachment and internalisation [32]. Images in Figure 5A show expression and co-localisation of both E-cadherin and β-catenin at the cell junctions of MCF7 and FLCARMCF7 cells. E-cadherin and β-catenin localisation appeared unaffected by incubation with Ad5eGFP at 3 minutes or 10 minutes (Figure 5). The intensity of E-cadherin (Figure 5A) and β-catenin (data not shown) at MCF7 and FLCARMCF7 cell junctions was also unaffected by incubation with Ad5eGFP. In order to confirm that Ad5eGFP bound to MCF7 and FLCARMCF7 cells, Ad5eGFP-Cy5 (Ad5eGFP labelled with fluorescent dye Cy5) was added for three minutes to coverslips that had been seeded with mixed population of MCF7 and FLCARMCF7 cells (1∶1 ratio) followed by fixation with 4% PFA. Figure 5B shows Ad5eGFP-Cy5 bound to MCF7 (identified by their lack of RFP expression) and FLCARMCF7 cells. Binding was more clearly visible in FLCARMCF7 cells, in keeping with their high CAR expression.

We then assessed the level of the E-cadherin/β-catenin complex in MCF7 and FLCARMCF7 cells to determine whether over-expression of CAR affected the formation of the complex. Figure 5C shows that E-cadherin is complexed with β-catenin and level of the complex is unchanged between the two cell types. This indicates that the over-expression of CAR does not affect the basal level of the E-cadherin/β-catenin complex. Moreover, addition of Ad5eGFP or bacterial expressed recombinant Ad5 fiber knob domain (FK) did not significantly alter the level of E- cadherin or β-catenin within the complex.

### CAR associates with the β-catenin complex

Previous studies demonstrated co-immunoprecipitation of β-catenin and CAR in A549 [18] and Sertoli cells [19]. Therefore, we sought to examine whether CAR also associates with β-catenin in FLCARMCF7 cells and whether such an association was affected by incubation with Ad5eGFP. Figure 5D shows that CAR co-immunoprecipitated with β-catenin, providing further evidence of direct or indirect interaction between CAR and β-catenin. Interestingly, incubation with Ad5eGFP increased the level of CAR associated with β-catenin up to 6 hours post infection (Figure 5D). Additionally, incubation with FK also increased the level of CAR associated with β-catenin (Figure 5D). For cells treated with Ad5eGFP, a significant difference was observed as early as three minutes post infection. One possible explanation for this observation is that binding of Ad5eGFP or FK to CAR induces CAR clustering and therefore increased association with β-catenin. Alternatively, the presence of Ad5eGFP or FK may increase CAR affinity for β-catenin or another as yet unidentified component of the complex. No association between CAR and β-catenin was seen in MCF7 cells, probably due to the very low endogenous levels of CAR found in these cells.

## Discussion

In the present study we demonstrate that Ad5eGFP increased paracellular permeability of MCF7 cells and CAR overexpressing MCF7 cells. This was independent of Ad5 replication as Ad5eGFP used in these experiments is replication deficient. Moreover, as these changes were observed early after the addition of Ad5eGFP to cells, we postulate that Ad5eGFP may disrupt cell junctions at an early stage of its infectious cycle, during virus binding and internalisation. This is consistent with previous studies showing Ad5 fibre-mediated increase in paracellular permeability of bronchial epithelium [18]. This could potentially provide a mechanism by which Ad5 can aid access to its own receptor (CAR) and therefore facilitate cell entry. As these experiments were performed in the presence of large excess of recombinant virus particles in non-polarised epithelial cells the significance of our observations in naturally occurring Ad5 infection cannot be determined. Interestingly however, loss of airway epithelial integrity by means of antibody-mediated disruption of E-cadherin function was shown to facilitate Ad infection, presumably by improved accessibility of CAR to incoming virus [33].

Here we also provide evidence that the addition of Ad5eGFP to MCF7 and FLCARMCF7 cells altered the molecular dynamics of E-cadherin. We assessed the dynamics of E-cadherin by measuring the rate of recovery of E-cadherin and the amount of fluorescent E-cadherin present after photobleaching MCF7 and FLCARMCF7 cell junction in the presence or absence of Ad5eGFP. It is important to note that these two measurements are not directly coupled and do not necessarily correlate. Regulation of E-cadherin levels at mature as well as immature cell junctions is a dynamic process. In immature and developing junctions mobile E-cadherin fraction is more abundant compared to mature cell junctions where the immobile fraction is in the majority [26], [27]. The mobile E- cadherin diffuses on the membrane and exchanges with the stable E-cadherin population at contact sites [26]. In Drosophila, a dynamic actin population controls E-cadherin movement across the membrane, whereas a separate population of actin molecules anchors and stabilises E-cadherin at points of cell contact [26]. Even in mature junctions there is considerable E-cadherin recycling between membrane and intracellular E-cadherin pools, a process dependent on vesicle trafficking and endocytosis [27]. It is interesting to note that changes in the half-life of E-cadherin recovery of the order observed in our studies have been correlated with cell migration in vitro [34].

Our observation that Ad5eGFP increased the mobile E-cadherin fraction and reduced cell surface E-cadherin levels in MCF7 cells indicates that in the absence of high CAR levels, less E-cadherin is stably associated with the cytoskeleton than in CAR overexpressing FLCARMCF7 cells junctions. Ad5eGFP also reduced the rate of E-cadherin recovery in MCF7 compared to FLCARMCF7 cells junctions, which suggests that E-cadherin recycling and movement occurs over a slower time period in the presence of CAR. The fact that Ad5eGFP increased paracellular permeability at MCF7 cell junctions may have been a consequence of the altered E-cadherin molecular dynamics in the presence of Ad5eGFP. Alternatively, the increased mobile E-cadherin fraction in the presence of Ad5eGFP could be secondary to disruption of cell junctions brought about by other means. Further studies are required to resolve these questions.

In FLCARMCF7 cell junctions we observed slower rates of E-cadherin recovery in the presence of Ad5eGFP, although this rate of recovery was faster in these compared to MCF7 cells. Interestingly, we also found that in the presence of Ad5eGFP, the mobile E-cadherin fraction was smaller and the immobile fraction larger in FLCARMCF7 than MCF7 cells. This would indicate that in the presence of high CAR levels (as in FLCARMCF7 cells) and Ad5eGFP, there is a shift towards more rapid turnover of E-cadherin to junctions, perhaps from a free cytoplasmic pool, with more E-cadherin being retained at FLCARMCF7 junctions as immobile fraction than in MCF7 cells, where CAR is expressed at low levels. These observations may help us understand why Ad5eGFP increased paracellular permeability to a greater extent in FLCAR than the parental MCF7 cells. It has already been shown that recombinant Ad fiber knob applied directly to the basolateral surface of well-differentiated airway epithelial cells can disrupt junctional complexes and increase paracellular permeability, probably by disrupting CAR-CAR homotypic interactions [18]. We therefore postulate that competitive inhibition of the CAR-CAR homophilic interaction in FLCARMCF7 cells by Ad5eGFP and its consequent disruption of CAR junctional complexes might account for this increased permeability and that changes in E-cadherin dynamics are in response to this. We speculate that CAR, when over expressed, may partially replace E-cadherin at junctions and in doing so alter the kinetics of E-cadherin recovery when cell junctions are disrupted. It is interesting therefore that we also observed that in the presence of over-expressed CAR there was reduced E-cadherin expression at FLCARMCF7 cell junctions. As reduced E-cadherin levels occurred without any changes to either the level or localisation of ZO-1, β-catenin or α-catenin it is unlikely that this is due to non-specific consequence of CAR over-expression. This conclusion is supported by recent studies that showed CAR expression in lung cancer cells was associated with absence of E-cadherin [35]. Such loss of E-cadherin at cell junctions did not alter paracellular permeability suggesting that CAR can compensate for E-cadherin in maintaining junctional integrity. Therefore, high levels of CAR may alter cell junction protein composition but not its integrity. This would be in keeping with previous studies that showed that CAR is required for initiation and maintenance of airway epithelial cell barrier and that CAR expression reduced paracellular permeability [36].

Our immunoprecipitation experiments showed increased association of CAR with β-catenin in the presence of Ad5eGFP. As recombinant Ad5 fiber knob had the same effect on the complex as Ad5eGFP, it is likely that the increased association with β-catenin is brought about by CAR interacting with its Ad5 ligand. One possible explanation for this observation is that binding of free FK or intact Ad5eGFP to the CAR extracellular domain promotes clustering of CAR and β-catenin so that they can interact. However, CAR clustering is thought to be a consequence of Ad5 but not FK binding to CAR. Indeed we have shown that Ad5 but not FK induces p44/42 dependent CAR dimerisation in cis in MCF7 cells [31]. An alternative explanation for our observations would be that Ad5eGFP or FK binding to CAR increases affinity of CAR for β-catenin. This raises the possibility that ligand-induced increased association of CAR with β-catenin could displace E-cadherin from the E-cadherin/β-catenin complex. It is interesting that it was previously suggested that CAR and E-cadherin do not directly interact and that CAR and E-cadherin may in fact compete for the same binding site on β-catenin [18]. The fact that E-cadherin/β-catenin complex level was the same in MCF7 and FLCARMCF7 cells basally and in response to Ad5eGFP would however suggest that it is unlikely that CAR and E-cadherin compete for binding to β-catenin in FLCARMCF7 cells. Our experiments do not exclude the possibility that CAR and E-cadherin interact directly. However, we have not consistently shown direct biochemical interaction between CAR and E-cadherin (data not shown) which would suggest that CAR and E-cadherin may not directly interact, or that they do so transiently thus not allowing detection of such interaction biochemically.

The mechanism by which CAR modulates E-cadherin basally and in response to Ad5 was not addressed in this study. Further studies are necessary to investigate this question. It is interesting in this context that JAM-A, a junctional molecule with which CAR shares homology, appears to negatively regulate E-cadherin levels in hepatocytes [37].

Our observation that CAR can modulate E-cadherin has broader implications beyond its potential role in Ad5 infection. This and the fact that CAR is a component of tight junctions raise the possibility that CAR contributes to the maintenance of epithelial cell junctions. Cell junctions are dynamic structures that assemble, break down and reassemble in response to a wide range of triggers such as cell division, cell migration, infection or inflammation. Understanding how CAR and E-cadherin interact at tight junctions will provide further insight on the pathways involved in the dynamics of cell junction formation and disruption.

## Methods

### Cells and materials

MCF7 (human breast cancer cells) were obtained from ATCC (ATCC number HTB-22™) and were maintained in Dulbecco's modified Eagle's medium (Gibco) supplemented with 10% fetal bovine serum (FCS, Sigma). MCF7 cells were transfected with plasmids containing FLCAR and cell clones (FLCARMCF7) were selected in the presence of geneticin (Gibco), as previously described [31]. Ad5eGFP was obtained from Vector Development Laboratory, Baylor College of Medicine, USA. 9000 virus particles (VP)/cell were used in all experiments. Ad5eGFP-Cy5 was Ad5eGFP labelled with Cy-5, a fluorescent dye producing a signal in the far-red region of the spectrum (670 nm). Ad5eGFP was mixed with 1 M NaHCO3 was added to one vial of Cy-5 and incubated for 30 minutes in the dark. The reaction was then terminated by the addition of 0.5 ml of 0.5 M Tris (pH 8). Excess dye was removed by dialysis using small dialysis cassettes (Pierce, 02203 ml) cassettes. Recombinant FK was produced and purified as previously described [3], [4], [38]. MCF7 and FLCARMCF7 cells were transiently transfected with E-cadherin GFP (gift of Dr Mark May University of Michigan).

### Generation of CAR constructs

FLCAR was amplified by PCR using the GCGCAAGCTTATGGCGCTCCTGCTGTGCTTCG (forward) and CATCGGCAAGCTGAATTCTACTATAGACCCATCCTTGC (reverse) primers to generate a 5′ Hind*III* and a 3′ Eco*RI* restriction site. The PCR product was then cloned into the pcDNA-RFP-C vector (a gift of Roger Tsien, UCSD, USA) to generate a C-terminal monomeric red fluorescent protein tag. All constructs were verified by sequencing prior to use.

### Western blotting and Immunoprecipitation

MCF7 and FLCARMCF7 cells were seeded on 10-cm plates in DMEM with 10% FBS and left for 4 hours. They were then washed twice with ice-cold phosphate-buffered saline (PBS) and lysed with 50 µl of RIPA buffer (10 mM Tris (pH 7.4), 150 mM NaCl, 1 mM EDTA, 1% Triton X-100, 1% sodium deoxycholate, 10 mM sodium fluoride, 1 µM okadaic acid with protease inhibitor complex). The cell lysates were then scraped into Eppendorf tubes and left on ice for 20 mins. Twenty micrograms of lysate protein was loaded onto 10% polyacrylamide gels and transferred to polyvinylidene difluoride membranes. Membranes were blocked for 30 mins at room temperature in TBS-T (Sigma) and 5% non-fat dry milk. Membranes were incubated with primary antibodies at an appropriate dilution in TBS-T, overnight at 4°C. The immunoblots were washed in TBS-T and incubated for 2 hours with horseradish peroxidase-conjugated anti-mouse or anti-rabbit immunoglobulin (Santa-Cruz Biotechnologies) at a 1∶5000 dilution. Immunoblots were then washed with TBS-T and visualized with ECL substrate reagent (Amersham Pharmacia). Membranes were then stripped and re-probed for total protein. Developed immunoblots were scanned in as tiff files and the protein bands quantified by densitometry with ImageQuant software. Bands of interest were normalised to the respective loading controls and the average percentage change over control (parental cells) levels quantified and plotted +/− SEM.

Immunoprecipitation (IP) was performed by combining 1×10^6^ cell equivalents in total volume of 200 ml of RIPA buffer with 5 mg of β-catenin antibody (Santa Cruz). The sample was then mixed at 4°C. After mixing, 50 µl of A/G beads (Santa Cruz) previously washed in 100 µl of PBS or lysis buffer 3× were added to the mixture and mixed at 4°C for 1 hour. The agarose beads were pelleted, washed in PBS and then resuspended in electrophoresis buffer. 25 µl of each sample was then run on a 10% PAGE gel. Gels were then subjected to Western Blotting and ECL detection.

### Cell adhesion assays

Reconstituted E-cadherin extra-cellular domain (R&D systems) was diluted using DPBS (PBS containing Ca^2+^) to a final concentration of 1.5 µg/ml or 5 µg/ml. The E-cadherin protein was then added to 4 wells of a 24-well plate at a volume of 400 µl/well and incubated for 60 minutes at room temperature. To control for non-specific adhesion control 300 µl of 1% BSA/PBS per well was used and as a positive control for adhesion, vitronectin at 10 µg/ml per well was used. Plates were then incubated for 60 minutes at room temperature. After incubation, excess unbound E-cadherin was removed and wells blocked with 300 µl/well 1% BAS/PBS for 30 minutes at room temperature to prevent any non-specific binding. MCF7 and FLCARMCF7 cells were disassociated from their flasks using 1 mM EDTA and resuspended in serum free media. Cells were then counted and 1×10^5^ cells added to each well followed by incubation for 2 hours at 37°C. After incubation, unbound cells were removed by washing with PBS. Adherent cells were then trypsinised until all the bound cells had detached; they were then counted using a haemocytometer.

### Confocal microscopy

MCF7 and FLCARMCF7 cells were plated out on coverslips and left to adhere. The cells were then washed with PBS and fixed. Cells were permeabilised with 0.1% Triton X-100-TBS for 10 minutes at RT. Non-specific binding was blocked using 5% BSA for 30 minutes at RT. 100 µl of each specified diluted antibody was added to each of the coverslips and left at RT for 3 hours. Following this the coverslips were washed and then incubated with goat anti-mouse Alexa Fluor 488 (Invitrogen). The secondary antibody was left on the coverslips for 1 hour at room temperature in the dark. The coverslips were washed once more before being mounted onto microscope slides using FluorSave™ Reagent (calbiochem). Cells were viewed using the LSM 510 META confocal microscope (Carl Zeiss Ltd). Images shown are single confocal slices unless otherwise stated.

### Analysis of junctional protein intensity

Confocal images of mixed populations (1∶1) of MCF7 and FLCARMCF7 cells immunostained for different junctional proteins were imported into Image J. Intensity levels of E-cadherin or β-catenin were analysed in the same image at junctions between FLCARRFP positive cells, or those without CAR and calibrated on a per pixel basis to correct for any differences in junction size/area. CAR-negative junction intensity values were normalised to 1 and all values for CAR positive junctions represented as a relative value to this. Values were pooled from multiple cells and images (n = >25 junctions per condition) over three independent experiments and represented as relative mean intensity.

### Cell permeability assay

MCF7 and FLCARMCF7 cells were grown to confluence on polyester transwell-clear filters (0.4 µm pore size, 12 mm diameter; Corning Costar Corporation). Ad5eGFP was added to the top chamber two hours prior to FITC-dextran addition (1 mg/ml) or simultaneously with fitc-dextran. 10 kD fitc-dextran (Sigma) was added to the top well and allowed to equilibrate for 1 hour. The fitc-dextran content of the lower chamber was then measured using a fluorescence plate reader.

### Fluorescence Recovery After Photobleaching (FRAP)

MCF7 and FLCARMCF7cells were transiently transfected in 24 well plates with E-cadherin-GFP or CAR-GFP for 24 hours before being plated on glass bottomed imaging chambers and maintained in growth media containing 25 mM Hepes. Confocal microscopy FRAP experiments were performed on a Nikon A1R microscope equipped with CFI Plan Fluor 40× oil objective. Images were captured, analysed and exported using NIS Elements AR software. Analysis of FRAP data was performed as described by Worth *et al* [39]. At least 3 images were taken every 10 seconds pre-bleach, and then a region of interest (ROI) was bleached (4×4 µm ROI used for all experiments), for 20 iterations with the bleach laser (488 nm) set at 100% and the pinhole at maximum. An image was taken immediately after the bleach and one 5 seconds after that, and then images were taken every 15 seconds for 5 minutes. Raw data was transferred to Excel and each cell corrected for photo-fading (by correcting for the amount the whole cell faded over the entire experiment) and resultant intensity values were converted into percentage values of the intensity pre-bleach. Corrected intensity values were plotted over time and the intensity at which the recovery curve plateau was defined. Mobile and immobile fractions were defined as the percentage recovery at plateau (mobile) and remaining non-recovered fraction at this time (immobile fraction). The half-life (τ1/2) of recovery was defined from corrected intensity data fitted to a single exponential equation and calculated using the equation *t*_1/2_ = ln 0.5/−τ. Data was pooled from at least 12 cells over 4 independent experiments.

### Statistical Analysis

All results were analysed for statistical significance using the SigmaStat software. Significance was determined by conducting a Two-Way or One-Way Anova where appropriate.

## Supporting Information

Figure S1 **Localisation of FLCARRFP in FLCARMCF7 cells.** MCF7 cells were transfected with RFP-tagged full length CAR (FLCARRFP). Confocal images taken in the red channel as well as the phase contrast images are shown for FLCARMCF7 (A) and parental MCF7 cells (B). FLCARRFP is shown at cell-cell junctions as well as intracellular compartments.
(TIFF)
