# Supplementary figures and images for: CAR Modulates E-Cadherin Dynamics in the Presence of Adenovirus Type 5
Source: PLoS One. 2011 Aug 5;6(8):e23056. doi: 10.1371/journal.pone.0023056 (PMC3151283; doi:10.1371/journal.pone.0023056)

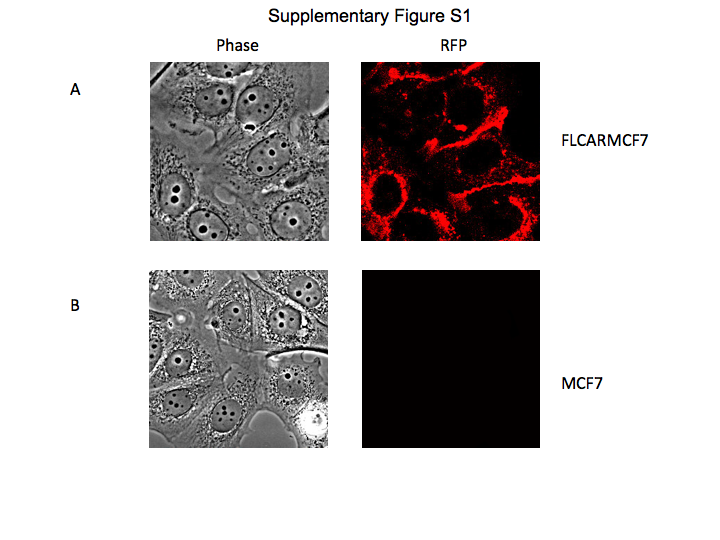

Supplement: Figure S1 — Localisation of FLCARRFP in FLCARMCF7 cells. MCF7 cells were transfected with RFP-tagged full length CAR (FLCARRFP). Confocal images taken in the red channel as well as the phase contrast images are shown for FLCARMCF7 (A) and parental MCF7 cells (B). FLCARRFP is shown at cell-cell junctions as well as intracellular compartments. (TIFF) [file pone.0023056.s001.tiff]
